# Do young people who self-harm experience cognitions and emotions related to post-traumatic growth?

**DOI:** 10.1016/j.jadr.2023.100683

**Published:** 2024-01

**Authors:** Alexandra Murray, Ruth Wadman, Ellen Townsend

**Affiliations:** a School of Psychology, University of Nottingham, NG7 2RD, United Kingdom; b Department of Health Sciences, The University of York, YO10 5DD, United Kingdom

**Keywords:** Self-harm, Post-traumatic growth, Recovery, Suicide, Interpretative phenomenological analysis

## Abstract

**Background:**

Adolescent self-harm is a vast public health concern with self-harm rates increasing each year. Looked-after adolescents are a group at increased risk of self-harm owing to adverse life-events and attachment issues, giving rise to difficulties with problem solving and relationship difficulties (Ogundele, 2020). Whilst research into risk factors of self-harm is plentiful, fewer studies have explored what factors might facilitate self-harm recovery. This paper investigates whether looked-after adolescents with experience of self-harm can experience cognitions and emotions related to post-traumatic growth (PTG).

**Methods:**

Secondary Data Analysis was conducted on a primary qualitative data set from a study including twenty-four looked-after young people aged between 14 and 21 with experience of self-harm; 20 females and 4 males. An Interpretive Phenomenological Analysis was conducted.

**Results:**

Five themes were identified, four of which shared similarities to prior research into the recovery of self-harm and manifestations of PTG: 1) Self-Reflection, 2) Communication, 3) Embracing and Appreciating Support, 4) Better Management of feelings. The fifth theme suggested a potential barrier to experiencing PTG; 5) Reliance on Self-Harm. Findings revealed four relevant themes suggesting that individuals who self-harm can indeed experience cognitions and emotions relevant to PTG.

**Limitations:**

This study utilised a homogeneous sample, limiting the generalisability of the findings to other populations. Perhaps future research should consider other populations of individuals who self-harm.

**Conclusions:**

We suggest there is a need for interventions for self-harm to focus on protective factors to increase PTG within an individual, potentially moderating the risk of suicide.

## Highlights

• Adolescent self-harm is vast public health concern that is on the rise.
• Looked-after adolescents are at significant risk of-self harm.
• Consideration of link between self-harm and PTG cognition.
• Focus on protective factors of recovery for self-harm

## 1 Introduction

It has been proposed that self-harm is a maladaptive coping mechanism often used when somebody struggles to manage emotional distress (Smith, 2016). Public Health England (2020) report over 100,000 hospital admissions each year in England as a result of self-harm. These figures are likely to be an underestimate as many individuals who self-harm do hide it owing to the stigma around self-harm being an attention-seeking behaviour, and patient's concerns around potential breaches of confidentiality if individuals do report self-harm (Rowe et al., 2014; Waller et al 2023).

Hawton et al., (2015) reported that the highest rates of self-harm are amongst adolescents aged 15-24. There was also a reported 22 % increase in self-harm rates within this age group between 2007 and 2016 suggesting that self-harm appears to be a major, and increasing, public health concern for adolescents. Adolescents with experience in the public care system are at increased risk of self-harm due to adverse life events; abuse, neglect, and family stresses (Harkess-Murphy et al., 2013; Evans et al., 2017; Ogundele, 2020). In this study, we focus on adolescents who are looked after (i.e., they are cared for by the public care system) and who self-harm, which is a very high-risk and under-researched group.

Repeated self-harm is a significant risk factor for suicide (Carroll et al., 2014; Beckman et al., 2018). World Health Organisation (2019) reported that suicide is the second most common cause of death for young adolescents globally. A large gap in our current understanding is whether individuals who self-harm can experience positive benefits following their self-harm behaviour. Earlier research has investigated the recovery process of individuals who self-harm (Kool et al., 2009; Ryan-Vig et al., 2019), but there is limited research into the potential positive aspects of recovery. Throughout these studies and this paper, recovery is defined as a subjective process whereby individuals who self-harm consider themselves as “recovered”, rather than recovery in terms of a medical remission of symptomology (Slade, 2010).

A few studies have explored the recovery process of individuals who no longer self-harm (Sinclair and Green, 2005; Kool et al., 2009). These studies found that individuals who self-harm required guidance and knowledge on how to manage their emotional wellbeing, and support to gain an understanding of their emotions and behaviours to aid their recovery. A phenomenological analysis conducted by Tofthagen et al. (2017) explored the recovery process of individuals who no longer self-harm. They found three themes of recovery: ``turning point'', ``coping with everyday life'' and ``valuing close relationships''. This research provides insight into potential recovery and continuance of self-harming behaviours as reported by those with lived experiences. It is important to note that existing research into the recovery of self-harm focuses on individuals who no longer self-harm describing retrospective recovery processes. This relies on individuals accurately reporting their experiences which may be influenced by individual memory and perception, impacting the credibility of such results. It is beneficial to explore individuals who do not perceive themselves as recovered and still experience self-harm, to explore whether they can experience any positive cognitions and emotions throughout self-harm periods which may aid their recovery.

Consistent with Slade's (2010) definition of recovery is the notion of post-traumatic growth (PTG). Park and Helgeson (2006) define PTG as positive outcomes experienced following an adverse experience. PTG is not a direct result of adversity but developmentally aids positive behaviours to help individuals overcome adversity through a more positive approach in line with a recovery process. Tedeschi and Calhoun (1996) proposed five manifestations of PTG that individuals experience in the face of adversity: ``a greater appreciation of life'', ``strengthening of relationships with others'', ``identifying new possibilities in life'', ``increased awareness of personal strength'' and ``spiritual growth''. PTG consists of a process whereby an individual's knowledge and acceptance of adversity improves well-being, as it offers insight and a sense of control to cope more positively, along with having a greater appreciation of relationships with others and life in general.

The current literature on PTG spans fields of research such as brain injury, cancer patients and sexual assault (Park and Helgeson, 2006). A study by Sheridan and Carr (2020) explored themes of PTG within individuals who had experienced institutional childhood abuse. Interpretative phenomenological analysis identified several themes suggestive of PTG within this population: ``the transformed self'' describing the development of positive self-impressions following a reflection of their experiences, and ``important others'' referring to participants' ability to form positive relationships and acknowledging the importance of discussing their life experiences. Findings suggest that individuals who experience childhood adversity can experience PTG (Woodward and Joseph, 2003; Tranter et al., 2021). This paper aims to explore whether PTG is relevant to individuals who self-harm in supporting their recovery process. This paper conceptualises PTG in relation to self-harm by exploring whether individuals who self-harm can experience cognitions and emotions related to Tedeschi and Calhoun (1996) manifestations of PTG. If there is relevance of PTG within self-harm, perhaps this could be useful to facilitate the recovery process of individuals from self-harm behaviour. However, it should be acknowledged that whilst self-harm can be traumatic in itself, it may also be used as a coping mechanism to cope with other traumatic events. Therefore, when exploring the relevance of PTG in individuals who self-harm, it is important to consider the possibility of other traumatic experiences in addition to their self-harm.

A recent study by Yasdiman et al. (2022) explored the protective function of PTG against entrapment and suicidal ideation. They found a negative correlation between PTG and suicidal ideation. However, they also found that PTG did not moderate the relationship between entrapment on suicidal ideation. Currently, there is no specific research evidencing that young people who self-harm can experience PTG-related cognitions and emotions. Therefore, this study aims to address this apparent gap in the literature. Although there appears to be minimal research advocating PTG as a protective factor against suicidal ideation within identified groups, studies predominantly focus on war veterans (Bush et al., 2011). This limits the transferability of the results to other individuals who self-harm. `

If PTG is relevant to individuals who self-harm, perhaps it may serve as a protective factor against self-harming behaviours which retrospectively would then facilitate a recovery process within these individuals.

This report describes a secondary qualitative analysis of an existing dataset from an interview study investigating individuals' experiences of self-harm using Interpretative Phenomenological Analysis. This report aims to explore whether looked-after adolescents who self-harm can experience cognitions and emotions related to post-traumatic growth (PTG), and whether experiencing positive outcomes may facilitate recovery and consequently moderate the risk of suicide.

## 2 Method

### 2.1 Participants

Participants in Wadman et al's. (2017a) study comprised of twenty-four adolescents who had self-harmed in the last six months and were looked after in the public care system (i.e. foster care, residential care); there were twenty females and four males. Eight of these participants were care leavers. Participants ages ranged from 14 to 21 years (M = 16, SD = 1.70). All participants were recruited in the East Midlands (UK) via several recruitment strategies. Eight participants were recruited through Child and Adolescent Mental Health Services (CAMHS), ten participants were recruited in the community (via a self-harm support organisation and wider advertising) and six participants were recruited through social care.

### 2.2 Design

In Wadman et al's. (2017a) study, participants were questioned via semi-structured interviews using an interpretative phenomenological approach. Their aim was to explore how individuals who self-harm report their experiences and perceptions of self-harm, and their views and experiences of support services. Participants were questioned about their first and most recent self-harm episodes. The interviews were conducted between March 2014 and April 2015 in private at home, university or a volunteer centre and lasted between 18 and 82 min long. Examples of questions asked in the study: ``Why do you keep on self-harming?'', ``Who supports you at the moment?'' and ``What might stop you from hurting yourself?'' Participants were also asked about their perception of the support services received to investigate if beneficial or not. The interviews were audio-recorded and transcribed.

In this paper, a secondary analysis was performed utilising data collected from Wadman et al. (2017a) study to explore if individuals who self-harm can experience cognitions and emotions related to PTG. This primary data source was deemed appropriate as all participants had encountered adversity, having looked-after status and self-harm episodes. Moreover, the questions asked in the interviews were likely to elicit thoughts and feelings related to recovery (e.g., What might help to stop self-harm?). This secondary data analysis adopted a ``new perspective focus'' strategy as outlined by Heaton (2003), as the data was explored with a different focus than the original study.

### 2.3 Analysis

Interpretative Phenomenological Analysis (IPA) was considered the most appropriate analysis for this paper as IPA explores how individuals interpret and make sense of their experiences. An IPA approach felt appropriate in exploring lived experiences of self-harm as the focus is on phenomenology; individuals' sense-making of their experiences. IPA acknowledges that individuals may struggle to interpret and express emotions in certain traumatic experiences such as self-harm. Smith (2011) reported guidelines to assist in conducting an IPA on a larger sample size, which this analysis adheres to. Other studies in the literature have been found to conduct an IPA on larger sample sizes which further supported this analysis decision (Vignoles et al., 2004; Tuohy and Cooney., 2019); Cooper et al., 2022).

Data was analysed by AM using Smith et al's. (2009) six stages to conduct an IPA. In the initial noting stage, any quotes of interest were noted as descriptive; describing the participant's experience, linguistic; focusing on the type of language used i.e., idioms, pauses etc., and conceptual level; moving away from explicit claims of the participant. In the developing emerging themes stage, the initial notes were reviewed to identify any commonalities. A process of abstraction was conducted to search for connections across themes to identify patterns between emerging themes and develop superordinate themes. Upon completion, a table was created displaying the abstraction process (see appendix). This table displays the emergent themes that were used to develop the superordinate themes. Consistent with Smith's (2011) guidelines for IPA with larger datasets the analysis was conducted at a group level rather than at an individual case level. The lead author (who undertook the analysis) had prior clinical experience as an Assistant Psychologist working in a rehabilitation unit for adults with an acquired brain injury and did not have any detailed knowledge or practical experience of working practices with looked-after young people who have experienced self-harm.

Five themes were identified in this analysis; two of which were present in at least twelve participants, and the other three were present in at least eight participants which corresponds to Smith's (2011) IPA guidelines for larger sample sizes (see Table 1).

### 2.4 Ethical approval

Ethical approval was already obtained by the original study from the department research ethics committee and the Social Care Research Ethics Committee (as part of the NHS Health Research Authority in England). Additionally, Tripathy (2013) highlighted that when conducting a secondary analysis, there is an ethical concern of breaching participant's confidentiality, if they can be identified in the dataset. However, as part of the conducted reviewing process ethical consideration was implemented by removing potentially identifying information.

## 3 Results and discussion

In line with the aims of this analysis and the larger sample size, narrative descriptions of the themes identified are presented within the context of cognitions and emotions relevant to PTG, illustrated by selected extracts from the participants’ interviews.

Five superordinate themes were identified: 1) Self-Reflection, 2) Communication, 3) Embracing and Appreciating Support, 4) Better Management of feelings and 5) Reliance on Self-Harm. The first four themes capture some relevance of thoughts and feelings relating to PTG for this population. The fifth theme denotes a potential barrier for this population to experience PTG-related cognitions and emotions. During analysis, a sixth theme of ``suicidal ideation'' (*N* = 7) was identified. However, this was discarded as Smith's (2011) guidelines outline a minimum of eight participants to demonstrate theme recurrence.

### 3.1 Self-reflection

Eleven Participants could identify what led them to engage in self-harm behaviour and understand why the ``problems really started'' (ID01) through a process of self-reflection. Reflection empowered participants to gain insight and awareness of their emotions in relation to their self-harm.

``Because I think, especially when I was younger with it, I couldn't, I didn't even know the words to express how I was feeling.'' (ID 01).

``I can understand why I was self-harming before. Whereas I used to, didn't know why, I didn't, I just had these feelings, like obviously looking back at the first incident, I can know that I was feeling angry but at the time I didn't know what angry was'' (ID10)

When describing earlier self-harm experiences and how this had changed over time, ID01 reflects on being less mature and unable to identify and express their emotions, whereas they could now acknowledge and understand why they self-harmed with associated feelings of anger. Undoubtedly, participants have been subject to extreme adversity and trauma, magnified by multiple care placements and several self-harm episodes. Therefore, this understandably impacts on an individual's ability to express emotions during complex periods. This highlights the importance of developing insight and understanding of experienced emotions and how these emotions can now be identified and linked to negative behaviours such as self-harming.

Reflecting on self-harm enabled participants to recognise that they are not alone, many individuals who self-harm ``hide'' their scars due to embarrassment (ID29) and because they think nobody understands ``I didn't think they'd understand'' (ID07). Through self-reflection, ID03 was able to overcome feelings of isolation which may have developed due to the stigma and shame surrounding self-harm due to a general misunderstanding of this behaviour.

``I don't [pause] single myself out as much as I used to [pause] cause it's more [pause] the thing is the thing is cause [pause] when you're new to it you don't realise how [pause] sort of [pause] how often it happens'' (ID03)

For these participants, being able to reflect on their self-harm experiences and gain more understanding assisted them in dealing with the difficult and intimidating nature of their self-harm behaviour. Engaging in a process of introspection and learning about their experiences of self-harm has also previously been reported as an important factor in facilitating the recovery of young adolescents who self-harm (Whitlock et al., 2009). This implies that although it may be challenging, reflecting and understanding personal experiences can support the recovery process of self-harm.

In relation to PTG, reflecting on adverse experiences shares similarities with Tedeschi and Calhoun's (1996) ``personal strength'' manifestation of PTG, as it implies an inner strength and willingness for individuals to understand their self-harm. Tedeschi and Calhoun (2004) proposed that individuals who experience PTG are better able to manage distressing emotions through a process of deliberative rumination. Therefore, highlighting that actively engaging in a process of self-reflection can facilitate PTG as this enables people to understand their experiences and develop personally.

### 3.2 Communication

Another positive aspect identified in ten participants was being ``more able to speak about it.'' (ID18). These participants reported being able to ``know who talk to'' (ID12) and recognised the importance of communication in developing relationships and supporting them to cope with their emotions.

``I told my care co-ordinator because I get on well with her, which I'm quite glad of. Which is quite nice because I didn't get on with her before and then when I got reassigned to her, I was like ``oh don't like her but I'll give her a go, I'll meet her for the first time and then ask to change'' But then I get on alright with her now (ID10)''

ID10 acknowledges the positive consequences of communication in improving relationships and building trust. Initially, ID10 had a pessimistic mindset towards their care coordinator, dismissing her before meeting and experiencing what she could offer, denoting an initial lack of hope in relationships. Despite this, ID10 describes now being more able to communicate, and an improved relationship with the care coordinator. It may be extrapolated that perhaps ID10 ``didn't get on with her before'' because prior qualitative studies have found that individuals who self-harm struggle to communicate and trust others (Klineberg et al., 2013; Wadman et al., 2017a).

The difficulties experienced with communication in this study appear relevant to Bowlby's (1979) attachment theory suggesting that a stable caregiver is vital to provide the foundations for individuals to develop future relationships. Participants have experienced multiple care placements and lack a stable caregiver to provide such foundations. Therefore, it is understandable that communication and developing relationships are challenging and it is remarkable that some individuals can overcome this by disclosing their self-harm ideation.

One participant appreciated the power of communication in preventing self-harm from escalating into ``something worse'' (ID24). ID24 describes observing the detrimental effects of self-harm in the hospital. Perhaps, ``seeing people in horrible states'' was a turning point for them to realise the importance of communication to prevent this from happening. ID24 describes communication as ``always'' being good, emphasising the significance of communicating every single time, and the importance of communication in preventing self-harm from escalating for this individual.

``I think it's always good to tell someone erm rather than it escalating into something worse cos like I said when I was in hospital I saw people that like I said I saw things that weren't very nice and I saw people in horrible states" (ID24)

Being able to disclose self-harm ideation has been reported in the literature as a significant issue for individuals who self-harm, preventing these individuals from accessing support (Owens et al., 2012; Singh, 2018). Similarities found in a quantitative study conducted by Townsend et al. (2016), ``I could not tell anyone how I was feeling'' was recognised as the second highest frequency item reported for both individual's first and most recent episodes of self-harm. Although, in this study some individuals were better able to communicate and recognise the importance of all communication.

Additionally, to overcome the challenge of verbally disclosing self-harm one individual (ID13) utilised a non-verbal way of communicating with staff/carers through a ``card system''. The ability to be creative and identify an alternative to talking highlights the importance of alternative communication methods for individuals. This also illustrates a determination for individuals to overcome perceived barriers preventing them from disclosing self-harm. Tedeschi and Calhoun (1996) suggest that ``personal strength'' and ``relating to others'' are essential to PTG as they empower individuals to express emotions and value the importance of having somebody to confide in.

``I think learning how to talk to people even if it's trying to find other ways, alternative ways of communicating with people when things happen rather than just talking in other ways, I had this card system, where I have a yellow card for keep an eye on me, and a red card for open my door and come into my room.'' (ID13)

Openly discussing traumatic experiences and sharing negative emotions has been reported as being psychologically beneficial and fundamental for individuals to experience PTG (Luszczynska et al., 2005; Henson et al., 2021). Similarly, the results evidence the significance of communication in individuals who self-harm as well as cancer patients bearing relevance to PTG.

### 3.3 Embracing and appreciating support

When asked about the support services they received, fourteen participants expressed a willingness to accept support to help them cope with their emotions: either professional support such as ``counselling'' (ID02) or ``family and friends'' support (ID21). However, before receiving support these individuals were unsure of whether support was available, and how this would benefit them ``I didn't know whether I was gonna get any help'' (ID07). This ability to now embrace and appreciate support reflects personal growth in these individuals through acknowledging that they need support and showing gratitude towards this support. This relates to Tedeschi and Calhoun's (1996) ``personal growth'' manifestation of PTG as it implies an inner strength within these participants to accept and embrace the support offered.

``I have the support I need through my friends and my family and I know that now, whereas before I didn't know.'' (ID21)

``I'm going to have a bit of counselling to try and [pause] sort out the granddad thing a bit more.'' (ID02)

ID21 describes now being able to identify the support they ``need'', whereas before they were unable to do so, implying a personal growth. Such support appears to be a necessity and essential to enable the individual to embrace and appreciate this provision. Arguably, without such support, self-harm may escalate. ID02 also uses the idiom ``weight off my shoulders'' to describe potential future benefits of counselling. The use of this idiom conceptualises self-harm as a burden of emotional distress providing constant trouble for individuals who self-harm. However, ID02 believes that by accepting support the distress will be reduced, and consequently they will no longer be burdened. Furthermore, this emphasises a required willingness to accept support and seek help for self-harming behaviours.

Participants also recognised positivity when embracing and accepting support as this helped with more positive coping mechanisms, and enabled them to display gratitude towards those who helped, e.g., (ID13) identified as ``the hospital staff''. This implies an informed appreciation from individuals for the support that is available and provided for them.

``So I thought ``okay I'm going to tell Xa,'' and Xa was like ``okay'' because I was very close to tears, I don't like crying but yeah. Xa took me to hospital, to A&E and I stayed in hospital for 3 weeks and then I went to X afterwards, and I stayed there for 6 months and then I came here. I got better and I came here. That's what happened. The hospital staff were amazing'' (ID13)

``They've actually like helped me like a lot more like if they see me crying now they'll come to my room and like check on me before they think I might self-harm or something so they'll come and check on me which will stop me'' (ID07)

The ability to accept support provided participants with the opportunity to observe and experience the benefits of having support. (ID07) described the positive outcomes of accepting help with an element of surprise, stating ``They've 'actually like helped me''. The use of ``actually'' implies that they did not expect the carers to be helpful or possibly they felt beyond help. Individuals who self-harm appear to have a negative perception of themselves, and support systems, and are unable to recognise the potential for both which may prevent them from seeking support.

Embracing and appreciating support also appears central to the recovery of self-harm. Research into the recovery of self-harm supports Tofthagen et al's. (2017) ``valuing close relationships'' theme which encompasses the importance of accepting support and developing relationships. Similarities are also shared by Tedeschi and Calhoun's (1996) ``strengthening of relationships with others'' manifestation of PTG. All of which recognise the importance of relationships with others and developing a network of support.

### 3.4 Better management of feelings

Smith (2016) described self-harm as a maladaptive coping mechanism to help individuals regulate their emotions. Nineteen participants expressed that they had found alternative coping mechanisms to manage their emotions such as ``poetry'' (ID12)/``art'' (ID16)/``music'' (ID09) implying that they had an improved ability to manage their feelings with adaptive strategies.

``Writing down your feelings'll probably let everything out. Because as I said before, I only self-harmed for letting everything out but with a pen and paper let everything out then.'' (ID12)

``I like going for walks, distraction, being with people helps, can help, because I'm a people and they make me happier. I think better, better ways than just forgetting help rather than self-harming.'' (ID13)

Generally, self-harm appears to provide these young individuals with a cathartic release of the emotions that they are unable to cope with (Shore, 1994; Bradley et al., 2018). This is acknowledged by (ID12) whose ability to utilise alternative methods to ``let everything out'' provided a substitute coping mechanism for emotional distress rather than self-harm. This shares similarities to previous research into the recovery of self-harm which identified coping behaviours relevant to assist in individual recovery and the process of self-harm (Gelinas and Wright, 2013; Tofthagen et al. (2017)).

Similarly (ID13) describes ``forgetting'' their self-harm ideation as an effective alternative to self-harm, allowing them to focus solely on the present and let go of their distressing cognitions. This links to prior research which has found a positive effect of dialectical behavioural therapy in reducing self-harm episodes (James et al., 2008). Ideally, this individual also acknowledges that dealing with the behaviour through alternative substitutes such as walking and talking provides more substantial management of self-harm than trying to forget. This implies a personal development within the individual as they identified a more positive coping mechanism to alleviate their emotional distress.

Three participants (ID02, ID19, ID24) also described how gaining self-awareness enabled them to prevent self-harm episodes by recognising the importance of support from others i.e., friends. They described actively seeking out time with friends to avoid situations which they could not cope with such as being alone. This evidences a remarkable ability within these participants to identify vulnerabilities and create change.

``I hang around with my mates and like they like, come around and then like every time I see them it doesn't make me think about the bad problems, I like have fun and like let my hair down for a bit and like chill out, so I don't end up thinking about it and then its always when I come home I'm always thinking about it when I'm not with them. So I try and use Facebook a lot so I can talk to them.'' (ID19)

(ID19) describes having friends around as providing an opportunity to ``let my hair down''. This highlights the significance of acknowledging perceived protection and freedom, as the distraction appears to suppress their self-harm thoughts. It seems that when they are alone these individuals struggle to control self-harm ideation which inadvertently prevents them from enjoying everyday life. Therefore, being able to identify situations that trigger self-harm ideation, and identifying alternative solutions to these would create and provide individuals with a safe escape from such detrimental thoughts, and offer opportunities for PTG.

One individual described being better able to manage thoughts of self-harm, ``I'm stronger than that [pause] I can get through this just by myself I don't need [pause] I don't need to take an overdose'' (ID06). ID06 explicitly states they do not ``need'' to overdose which may imply a reduced dependency on self-harm and evidence more self-control, awareness and better management of their feelings.

The reflections above all point to a capacity to develop positive coping strategies about negative feelings, which could facilitate a recovery process. Having a ‘better management of feelings’ would also link to Tedeschi and Calhoun's (1966) ``personal strength'' conceptualisation of PTG. Self-harm was described as providing individuals with short-term relief from emotions rather than letting these intense feelings manifest. Nevertheless, individuals acknowledged over time that they could also identify alternative behaviours to alleviate these feelings and prevent them from engaging in self-harm. These findings are compatible with Linley and Joseph's (2005) model of PTG which stipulates that coping strategies can assist PTG by reducing stress. Therefore, it may be that such coping strategies could facilitate PTG in individuals who self-harm. To further support the relevance of alternative coping strategies to the notion of PTG, Kunz et al. (2018) reported a relationship between positive coping strategies and high levels of PTG in spinal cord injury patients.

### 3.5 Reliance on self-harm

Although some participants used alternative coping strategies to manage their emotional distress more adaptively, nine participants reported that these substitute strategies e.g., music/poetry only provided a mere delay of the inevitable self-harm behaviours as ``the thoughts are all there'' (ID01). Prior research conducted by Wadman et al., (2017b) also reports difficulties with self-harm cessation in young adults, captured by the theme 'not believing they will stop completely'.

``it's an addiction really…I don't feel the same when I haven't self-harmed. Like I feel, I feel like stuff is building up all the time. (ID23)

``Erm… because it's addictive, I like the pain of it I suppose; I like the feel of it. And it's something that's so the norm to me now, to suddenly let go of it, it's quite daunting. If I've not cut in quite a long period of time, then I think to myself I need to cut. Because I'm scared of letting it go, that sounds really silly. But yeah, no I think that's why. It's just so addictive and it's such a comfort thing.'' (ID11)

ID11 expresses ``liking'' the physical pain of self-harm, as it relieves them from their emotional suffering. This reiterates the complexities of self-harm as the individual acknowledges the perceived gains from self-harm behaviours, which then reinforces the purpose and reliance on this behaviour making it difficult to cease. Additionally, individuals who self-harm are faced with further barriers as the idea of stopping can be ``daunting'', the behaviour becomes part of a perceived identity, control and something they then rely on. This emphasises the habitual nature of self-harm and how individuals are then unable to overcome what they distinguish as a behavioural addiction thus providing them with ``comfort'' by alleviating distress.

Self-harm was also described as an ``addiction'' or ``habit'' in which participants became reliant on to cope with their emotions. This highlights an additional barrier to individuals developing and experiencing PTG as addiction becomes another encompassing layer to be addressed and removed. Participants described knowing that self-harm provides them with instant relief from their emotional distress that is ``building up all the time'' (ID23). Self-harm has been reported within the literature as an addictive, habitual phenomenon that individuals struggle to overcome (Sutton, 2005).

``I see it more as an addiction it's not something that's going to stop, I mean I don't know I mean [beep sound] maybe I don't do it as worse as I used to but sometimes I do it just like I said it just depends'' (ID24)

Although several individuals were able to identify better ways to manage self-harm, it appeared that for some individuals this provided a temporary escape from self-harm. ID24 recognises that although they can better manage self-harm through substitute techniques, this only acts as a mere delay of self-harm which is ``not something that's going to stop''. Although these individuals identify alternative coping techniques, these techniques do not ``feel the same'' (ID23), which arguably increases the reliance individuals have on self-harm. Blasco-Fontecilla et al's. (2016) addictive model of self-harm which equates the emotional state preceding self-harm to withdrawal symptoms experienced by drug users would be relative. Their analogy of self-harm with drug addicts emphasises the dependency individuals have with self-harm. Sadly, both phenomena appear to be stigmatised in society, therefore it may be argued that if individuals who self-harm, refer to the perceived label as being an addiction, perhaps it may be more accepted and understood as a concept in society than self-harm appears to be. Furthermore, it creates more understanding, support and willingness to access what is available.

It is important to note that six out of the nine individuals who expressed a reliance on self-harm, were also in the ``better management of feelings theme''. This could suggest some evidence of PTG as they are beginning to manage their feelings. However, it must be acknowledged that substitute behaviours do not provide sufficient satisfaction to completely cease self-harm behaviours due to the positive reinforcement they gain from this. Additionally, ID03, ID05 and ID08 appear fully reliant on self-harm and did not report any better management of emotional distress. The most recent episodes of self-harm within these individuals being one-two weeks ago, which reflects the heterogeneity among individuals about PTG and being able to gain control over self-harm urges.

PTG and recovery are two processes that are unique to each individual as experiences and progress differ. Although individuals can utilise alternative ways of coping with self-harm ideation, there still appear to be restrictions on the ability to fully recover because of perceived reliance. Although strategies and support may be similar, the complexities of individuals are unique and should be assessed to gain mutual awareness of the support required to further develop PTG. While the paper has identified alternative techniques as temporary distractions that benefit individuals, one cannot minimise the underlying emotional distress, which requires further intense interventions and support. Alternative positive behaviours to self-harm are part of the process and as evidence, they can provide some immediate benefit to some individuals.

### 3.6 Suicidal ideation

Although the ``Suicidal ideation'' theme was removed from the analysis, it is considered important to not dismiss as the paper found that some individuals who show signs relevant to PTG still reported wanting ``to die'' (ID04). This implies that although individuals who self-harm may show signs relevant to PTG, this did not alleviate their suicidal ideation. This finding adds to Yasdiman et al (2022) research who found a negative association between PTG and suicidal ideation as this research suggests that although there may be some relevance of PTG for individuals who self-harm, they still remained with suicidal thoughts.

Owens et al. (2005) investigated help-seeking and primary care consultations before suicide and observed positive and negative signals in individuals who died by suicide. In contrast to Tedeschi and Calhoun's (1996) manifestation of PTG, “greater appreciation of life”, this paper supported the notion that individuals who self-harm can experience some positive outcomes in line with PTG whilst still experiencing suicidal ideation.

Reflecting on O'Connor and Kirtley's (2018) IVM model of suicide which focuses on risk factors of suicide, one may wonder if it would be more beneficial to look at potential PTG factors in individuals who self-harm taking into consideration self-harm is a significant risk factor for suicide. This may further assist individuals who self-harm to experience positivity, rather than focusing on risk factors. Perhaps there is a need to develop interventions to improve communication skills, and relationships, and supporting people to identify and express emotions which could be crucial in preventing suicidal ideation. Perhaps PTG could act as a moderator for self-harm, indirectly preventing suicidal ideation.

## 4 Limitations and future research

Although the analysis illuminates the relevance of PTG in individuals who self-harm, limitations should be considered. Firstly, the sample used was homogeneous, as they were all young adolescents who had a history of self-harm and experience with the public care system. Smith (2009) highlighted that qualitative findings in specific homogenous samples are advantageous for providing rich and descriptively deep analysis that can be transferred to other similar adolescents. However, it may be useful for future research to consider whether other populations of individuals who self-harm can experience cognitions and emotions related to post-traumatic growth. A recent systematic review of self-harm conducted by Troya et al. (2019) identified older adults to be another population for research interest in self-harm.

Additionally, secondary analysis was beneficial as it provided insight into the relevance of PTG in self-harm individuals with the potential for future research to investigate further. However, no themes were identified similar to Tedeschi and Calhoun's (1996) ``greater appreciation of life'' or ``spiritual growth'' manifestations of PTG, perhaps due to the constraints of secondary analysis. Additionally, this was also restricted by questions asked in the primary study. Questions could have been more specific to PTG such as ``Can you describe any positive changes you have experienced as a result of self-harm?'' which may have provided more credibility and insight into individuals who self-harm and experience PTG. Alternatively, participants could have been assessed using Tedeschi and Calhoun's (1966) PTG inventory; a 21-item scale to assess PTG across their five manifestations. This may have provided a more well-rounded insight into individuals who self-harm and can experience full PTG. Future research could be conducted on a primary data set using the PTG Inventory which may enable the researcher to ask follow-up questions, clarify participant's responses and pursue other avenues.

Jayawickreme and Blackie (2014) conceptualised PTG as a positive personality change which the study was unable to investigate due to secondary analysis. Future research may expand this further to ascertain if individuals who self-harm can experience PTG and may consider the personality traits of these individuals. Using an alternative method to the PTG inventory to assess PTG, such as the experience sampling methodology by Conner et al., (2009) may be more beneficial to investigate this, and understand PTG as a personality change. In this longitudinal method, participants are required to report current thoughts, feelings, and behaviours on multiple occasions over time, via an electronic device. This methodology seems appropriate for future research investigating the relevance of PTG to self-harm individuals over a period.

The findings of this paper highlight the significance of research into recovery and PTG, and the relevance of both in the process of recovery for individuals who self-harm. Therefore, this research will be relevant to research into interventions for individuals who self-harm. Perhaps such interventions could be focused more on PTG manifestations rather than risk factors to facilitate recovery. Haroosh and Freedman (2017) stipulate that PTG is a mechanism interlinked with recovery, as they share common positive changes, and PTG also maintains the recovery of individuals. The findings from this paper infer that individuals who self-harm were able to gain some skills as a result of their self-harm experience baring relevance to PTG, which contributed to their recovery process. Perhaps, future research could consider the relationship between recovery and PTG, to identify if one facilitates the other or if PTG could be considered a form of recovery.

## 5 Conclusions

This paper suggests that there is potential for individuals who self-harm to experience cognitions and emotions related to PTG. There are implications that perhaps self-harm interventions should focus on protective factors and training aspects of PTG to aid the recovery process and mitigate suicidal risk. Future research using prospective longitudinal methods on a primary data set may prove productive as both recovery and PTG are processes that change over time. Thus, enabling an individual's progress to be monitored more purposefully by measuring outcomes. Future research may utilise relevant measurements of PTG to investigate if a comprehensive evaluation of this development would be more relevant to different populations of individuals who have benefited and compare findings and strategies to those who self-harm.

## CRediT authorship contribution statement

**Alexandra Murray:** Methodology, Formal analysis, Writing – review & editing. **Ruth Wadman:** Data curation, Resources, Supervision. **Ellen Townsend:** Conceptualization, Supervision, Project administration.

## Declaration of Competing Interest

The authors declare that they have no known competing financial interests or personal relationships that could have appeared to influence the work reported in this paper.

## Appendix Supplementary materials

Image, application 1
