# Supplementary material for: Do young people who self-harm experience cognitions and emotions related to post-traumatic growth?
Source: J Affect Disord Rep. 2024 Jan;15:100683. doi: 10.1016/j.jadr.2023.100683 (PMC10801152; doi:10.1016/j.jadr.2023.100683)
Supplement: Supplementary file 1 [file mmc1.docx]

# Appendices

Appendix A

| Table 1  *Abstraction process to develop superordinate themes from patterns identified between emergent themes.* | |
| --- | --- |
| Superordinate theme | Emergent themes |
| Self-reflection | - *Identifying their weaknesses on reflection i.e. being unable to express emotions* - *Being able to think about their self-harm history and gaining understanding of what led to their self-harm* - *Overcoming not being able to express their feelings* - *Assessing their younger self in comparison to now* - *Thinking about consequences of self-harm* - *Able to recognise what helps them* |
| Communication | - *Knows who they can confide in and talk to* - *Talking as a helping function to the individual.* - *Sense of improved relationships through being able to communicate* - *Talking being important to the individual* - *Recognising positivity from talking* - *Being more confident in disclosing self-harm ideation* - *Identifying non-verbal form of communication to access help* |
| Embracing and Accepting Support | - *Able to identify sources of support; family/friends/counsellors.* - *A willingness to accept support* - No signs of resistance when support is put in place. - *Recognises positive outcomes from support* - *Acknowledges that it is okay to need help* - *Beforehand being unable to identify support.* - *Increased awareness of who supports them* |
| Better management of feelings | - *Identifying alternatives* - *Distracting themselves from self-harm ideation* - *Confidence in ability to prevent self-harm* - *Identification of triggers for their self-harm* - *Actively avoids situations that could lead to self-harm* - *Willing to put off self-harm urges* - *Recognising the positive impacts of alternatives* - *Increase in personal strength to manage self-harm* - *More control over self-harm* |
| Reliance on self-harm | - *Addictive nature of self-harm* - *Habitual nature being problematic* - *Positive reinforcement of self-harm* - *Self-harm being the norm* - *Feeling a need to self-harm* - *Fear of letting go* |
| Suicidal Ideation | - *Thinking about suicide* - *Self-harm as a means to suicide* - *Wanting to take their life* - *A desire to no longer live* - *Hopeless for the future* - *Pessimistic mindset.* |
